# Management of Orthodontic-Induced Gingival Enlargement: A Case Report

**DOI:** 10.7759/cureus.47660

**Published:** 2023-10-25

**Authors:** Unnati Shirbhate, Pavan Bajaj, Dhruvi Solanki, Sneha Dare, Swayangprabha Sarangi

**Affiliations:** 1 Department of Periodontics, Sharad Pawar Dental College, Datta Meghe Institute of Higher Education and Research, Wardha, IND; 2 Pediatric and Preventive Dentistry, Sharad Pawar Dental College, Datta Meghe Institute of Higher Education and Research, Wardha, IND; 3 Conservative Dentistry and Endodontics, Sharad Pawar Dental College, Datta Meghe Institute of Higher Education and Research, Wardha, IND

**Keywords:** orthodontic induced gingival enlargement, gingivoplasty, gingivectomy, scalpel, orthodontic therapy, gingival enlargement

## Abstract

The aetiology of gingival enlargement (GE) is highly distinct. Plaque-induced gingival inflammation can be the sole reason for gingival enlargement. Poor dental hygiene, irritation from anatomical variations, and ineffective restorative and orthodontic appliances are all factors that encourage the formation and retention of plaque. In the given case report, a case of gingival enlargement associated with an orthodontic appliance of a 23-year-old female patient referred from the Department of Orthodontics was reported to the Department of Periodontics. Under local anaesthesia, the excess gingival tissue is removed using a scalpel by gingivectomy and gingivoplasty procedures. The gingivectomy and gingivoplasty procedures using a scalpel gave the best results in the orthodontic treatment associated with gingival enlargement. After achieving hemostasis, the periopack (Coe-pack) was placed to assist healing by protecting the tissue. The above case report can appreciate the gingival tissue covering almost half of the crown, causing plaque retention and presenting the patient with aesthetic concerns. After the surgical procedure, a proper gingival contour eliminates suprabony pockets and provides pleasant esthetics. This case report demonstrates that eliminating the suprabony pockets by gingivectomy and gingivoplasty leads to a physiologic gingival contour and eliminates plaque retention. The conventional scalpel gingivectomy procedure is an effective form of treatment when indicated.

## Introduction

The aesthetics of the gingiva surrounding the teeth in the anterior maxillary region of the mouth are affected mainly by their appearance. The symmetry and form of gingival tissues can substantially impact the harmonious appearance of the natural or artificial dentition. Patients' increased demand for more aesthetically pleasing outcomes may influence their treatment choice. An ideal anterior appearance is provided by healthy periodontal tissues devoid of inflammation [1]. An increase in the size of the gingiva is the hallmark of gingival enlargement (GE), also known as gingival overgrowth. Accurately identifying the cause of the enlargement is necessary for effective management [2]. Fixed orthodontic equipment and persistent periodontal diseases may be related. Increased plaque accumulation and ineffective dental hygiene practices lead to gingival enlargement. Throughout their orthodontic treatment, individuals experience GE due to this mechanism, but its exact nature is unknown. The equilibrium between the microbial agent and the host's immune responses determines periodontal disease's initial stage and development [3]. Plaque buildup and the colonisation of significant periodontopathic bacteria surrounding the retentive components attached to the surface of teeth are affected by the presence of fixed orthodontic appliances. Some causative factors for GE caused by orthodontic treatment were discussed by Kloehn and Pfeifer, including mechanical irritation from bands, chemical irritation from cement, food buildup, and poor oral hygiene [4]. Several different etiologic factors might contribute to gingival enlargement. Gingival enlargement may have one reason, which is plaque-induced inflammation. Poor dental hygiene, discomfort from anatomical variations, and ineffective restorative and orthodontic appliances are all factors that favour plaque buildup and retention [3,4]. The periodontal tissue must be completely free of these harmful substances to heal. However, when GE is widespread and self-oral hygiene procedures are hampered, nonsurgical periodontal therapy, such as scaling and oral hygiene instruction, is ineffective [5]. Each type of enlargement has a different treatment regimen. Surgical and nonsurgical treatments are frequently combined, depending on the patient's needs. It is essential to keep in mind both functional and aesthetic requirements. Treatment is required due to the enlarged gingiva's unattractive appearance. This leads to gingivectomy, or the removal of overgrowth. The soft tissue wall of a diseased periodontal pocket is removed during a gingivectomy. Scalpel, laser, electrosurgery, and chemosurgery are all techniques used during gingivectomy surgeries [6].

## Case presentation

**Figure 1:**
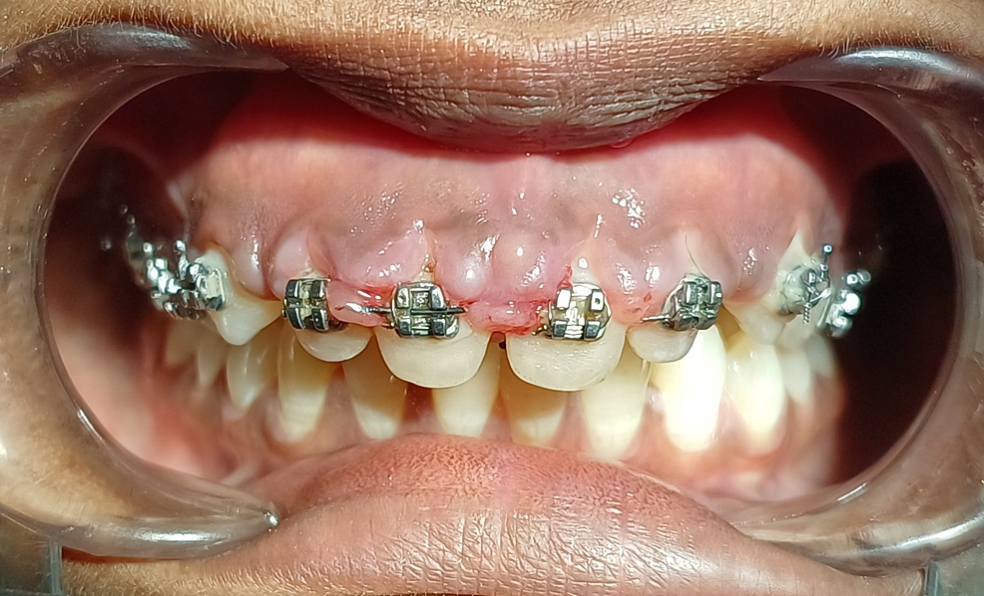
Pre-operative view showing orthodontic-induced gingival enlargement in anterior esthetic region.

A case of gingival enlargement caused by orthodontic therapy affecting a 23-year-old female patient referred from the Department of Orthodontics and reported to the Department of Periodontics is described in the case report. Additionally, it was noted that the patient could not maintain good oral hygiene when the GE became noticeable. During an intraoral examination, it was found that there was GE in the maxillary anterior region. As seen in Figure 1, there was significant inflammation at the interdental papilla and marginal gingiva and generalised bleeding on probing.

**Figure 2:**
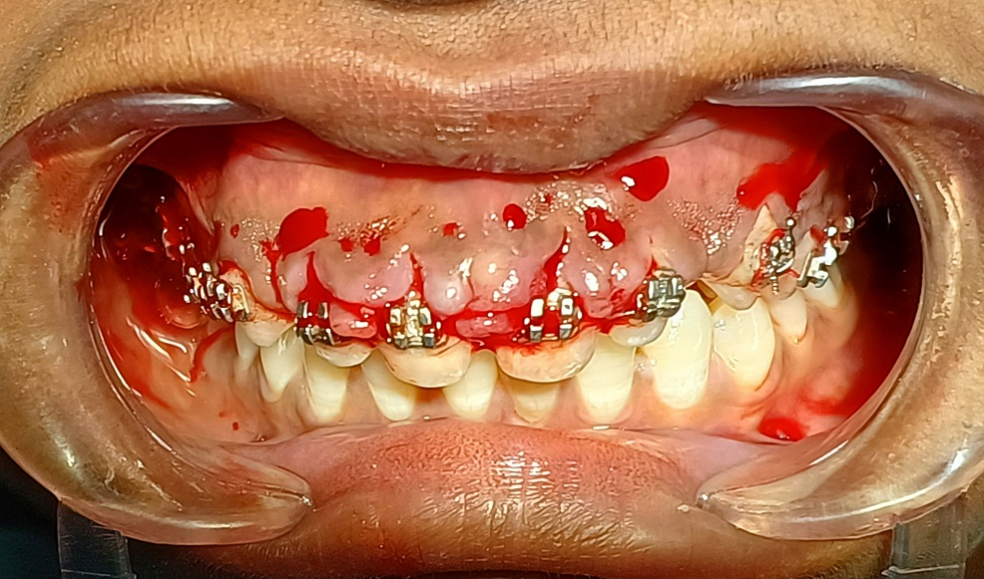
Bleeding points were marked till the pocket depth by using pocket marker.

The gingiva was generally consistent and firm, with some loss of contour and stippling. It became apparent that the overall pseudopocket measured 4-5 mm. Considering the above findings, the patient is advised for initial therapy, surgical therapy, and a haematological examination. Scaling and polishing are done during the initial treatment and recalled after seven days. Haematological findings for haemoglobin level, bleeding time, clotting time, and random blood sugar level were done, which were within the normal range. Under all aseptic conditions and local anaesthesia, the bleeding points were marked by a pocket marker shown in Figure 2 with the maxillary anterior aesthetic region from the right maxillary second premolar to the left maxillary second premolar.

**Figure 3:**
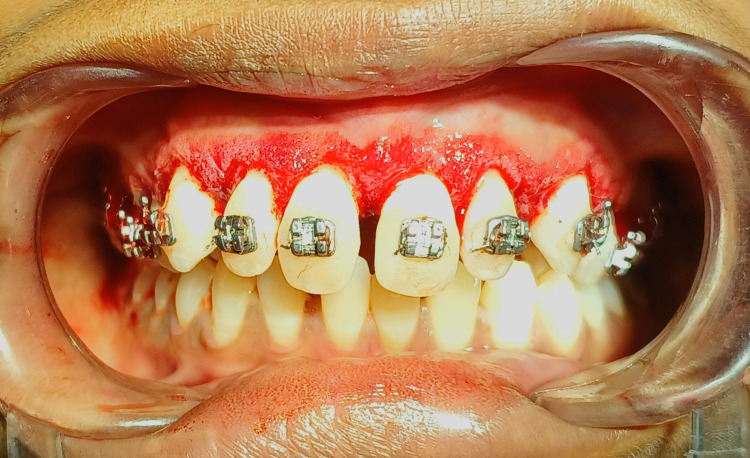
Gingivectomy and gingivoplasty performed by using conventional scalpel technique.

After seven days after achieving initial therapy, excess gingival tissue is removed using a scalpel through gingivectomy and gingivoplasty procedures. The gingivectomy and gingivoplasty procedures using a scalpel gave the best results in the orthodontic treatment associated with gingival enlargement, as appreciated in Figure 3.

**Figure 4:**
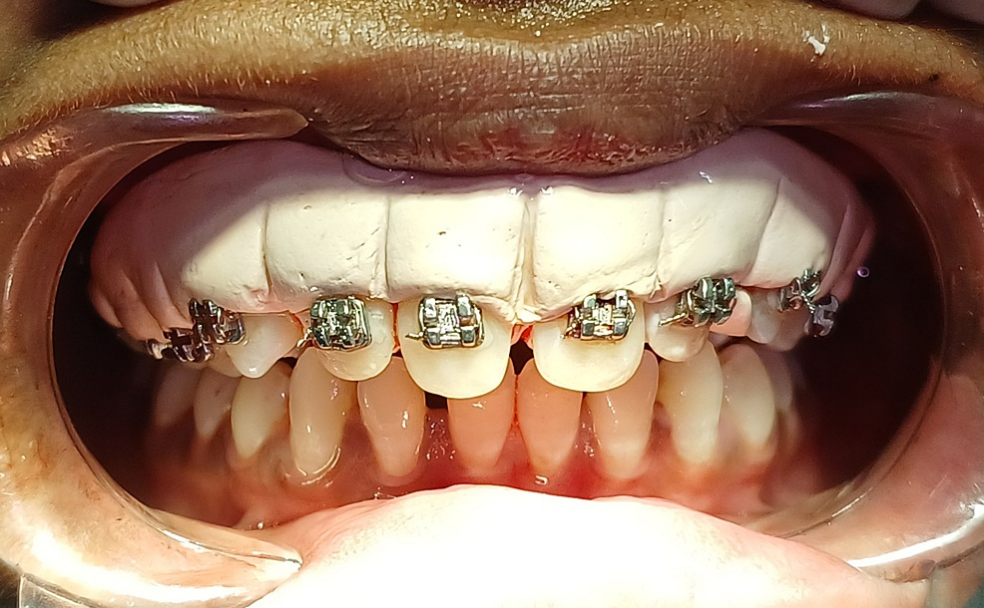
Periodontal pack is administered after the surgery.

After achieving hemostasis, the periopack (Coe-pack) was placed to assist healing by protecting the tissue, as seen in Figure 4.

**Figure 5:**
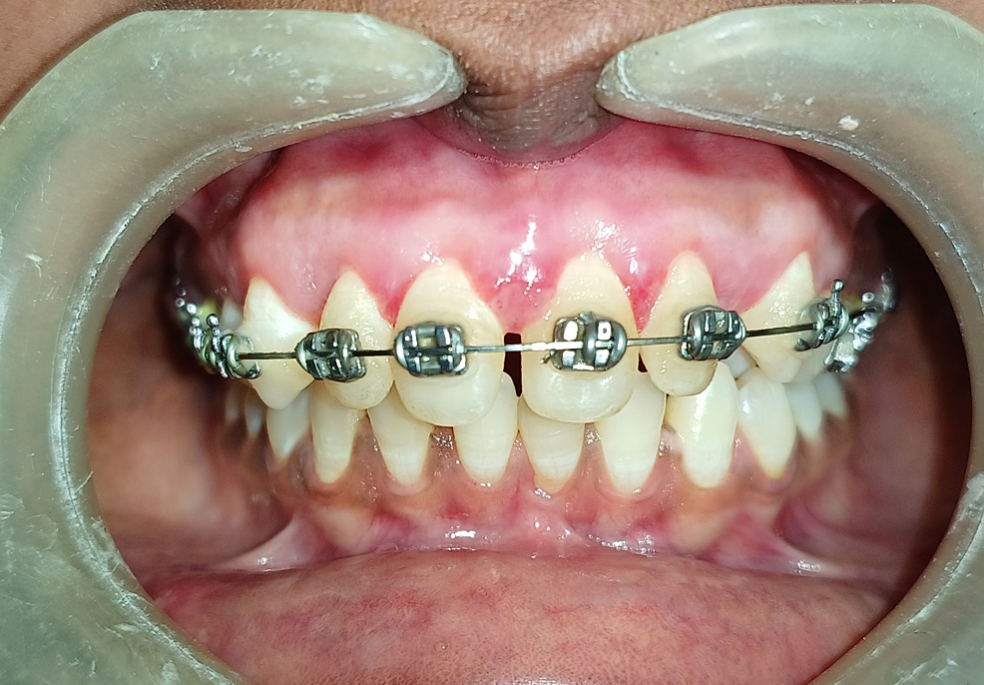
Post-operative view after seven days reveals satisfactory healing, proper gingival contour and improved esthetics.

**Figure 6:**
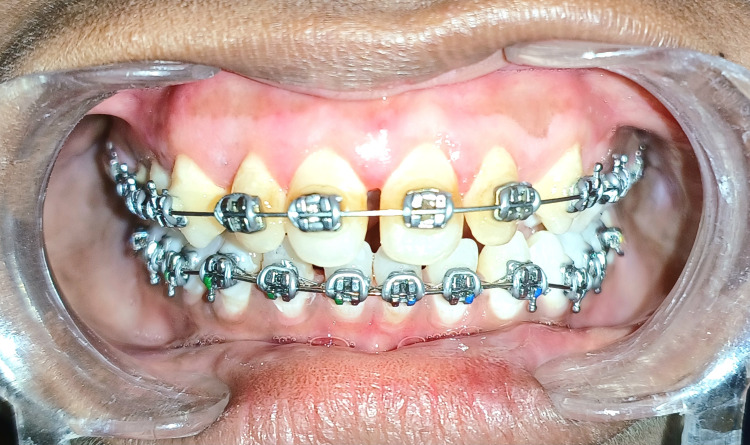
Follow up examination after two months reveals complete recovery in terms of healing of gingival inflammation.

The above case report can appreciate the gingival tissue covering almost half of the crown, causing plaque retention and presenting the patient with aesthetic concerns. After the surgical procedure, the patient was recalled for periopack removal after seven days. This shows that a proper gingival contour eliminates suprabony pockets, provides satisfactory healing, and provides pleasant aesthetics, as seen in Figure 5. The patient was recalled after two months for a follow-up examination, which revealed complete healing and a satisfactory outcome in terms of gingival inflammation after treatment, as shown in Figure 6. This case report demonstrates that eliminating the suprabony pockets by gingivectomy and gingivoplasty leads to a physiologic gingival contour, improves aesthetics, and eliminates plaque retention.

The patient was recalled after two months for a follow-up examination, which revealed complete healing and a satisfactory outcome in terms of gingival inflammation after treatment, as shown in Figure 6. This case report demonstrates that eliminating the suprabony pockets by gingivectomy and gingivoplasty leads to a physiologic gingival contour, improves aesthetics, and eliminates plaque retention.

## Discussion

The maintenance of oral hygiene is more challenging for patients with gingival overgrowth. The most common therapy for gingival overgrowth is still a surgical procedure; such treatment is advised when the overgrowth is severe. It entails electrosurgery, scalpel gingivectomy, overgrowth flap surgery, and laser excision [7]. In general, poor periodontal health among orthodontic patients has been linked to favourable conditions for plaque stagnation and difficulty performing standard oral hygiene procedures. According to Pinto et al., the incidence of GE arises as orthodontic treatment time increases [8]. Al-Abdaly et al. concluded that gingival index and gingival enlargement index, salivary flow, and pH salivary values, as well as predictive values for inflammatory gingival enlargement and its therapy, can be used to observe the severity of inflammatory gingival enlargement during orthodontic treatment [9]. According to Zanatta et al., orthodontic treatment participants are likelier to experience anterior gingival hypertrophy. They also found an association between excess resin around brackets and proximal anterior gingival bleeding [10].

Due to the prolonged nature of the treatment, gingival enlargement is frequently caused by the patient's orthodontic care. Careful instruction regarding oral hygiene and surgical technique is part of the treatment strategy for gingival overgrowth [1]. Before beginning orthodontic treatment, gingival and periodontal health needed to be assessed. Clinicians should be able to evaluate a patient's oral hygiene maintenance skills both before and during orthodontic treatment [8]. During orthodontic therapy, inflammatory gingival enlargement refers to a localised or generalised growth of gingival tissues extending to the gingival margin, interdental papilla, and attached gingiva. In persistent cases with fixed orthodontic appliances, acute or chronic inflammatory gingival enlargement is readily seen. Moreover, orthodontic therapy modifies the oral cavity's ecological features, which limits saliva's properties [9]. Although the precise process underlying the development of GE is unknown, it most likely involves enhanced fibroblast production of an amorphous ground substance containing a high concentration of glycosaminoglycans. Excessive epithelial cell proliferation and the development of GE may be significantly influenced by increases in type I collagen mRNA expression and up-regulation of keratinocyte growth factor receptors [10].

## Conclusions

Gingival enlargement is of prime concern to the patient as it impairs function and aesthetics. In excessive orthodontic enlargement cases, a properly timed surgical procedure to reduce the tissue to a normal contour will yield maximum benefit to the patient, reducing the number of clinical visits needed and improving the patient’s quality of life. This case report suggests that surgical periodontal treatment with the scalpel gingivectomy can effectively manage gingival health problems in patients with fixed orthodontic appliances. This demonstrates that eliminating the suprabony pockets by gingivectomy and gingivoplasty leads to a physiologic gingival contour, eliminates plaque retention, and gives the patient an aesthetic appearance. Periodic periodontal maintenance care during orthodontic therapy would avoid such consequences and preserve gingival health.
